# 3-Phosphoinositide-dependent Protein Kinase-1 (PDK1) promotes invasion and activation of matrix metalloproteinases

**DOI:** 10.1186/1471-2407-6-77

**Published:** 2006-03-21

**Authors:** Zhihui Xie, Hongyan Yuan, Yuzhi Yin, Xiao Zeng, Renkui Bai, Robert I Glazer

**Affiliations:** 1 Department of Oncology, Georgetown University School of Medicine, and Lombardi Comprehensive Cancer Center, Washington, DC, USA; 2 Laboratory of Allergic Diseases, National Institute of Allergy and Infectious Diseases, Rockville, MD, USA; 3 Department of Molecular and Human Genetics, Baylor College of Medicine, Houston, TX, USA

## Abstract

**Background:**

Metastasis is a major cause of morbidity and mortality in breast cancer with tumor cell invasion playing a crucial role in the metastatic process. PDK1 is a key molecule that couples PI3K to cell proliferation and survival signals in response to growth factor receptor activation, and is oncogenic when expressed in mouse mammary epithelial cells. We now present evidence showing that PDK1-expressing cells exhibit enhanced anchorage-dependent and -independent cell growth and are highly invasive when grown on Matrigel. These properties correlate with induction of MMP-2 activity, increased MT1-MMP expression and a unique gene expression profile.

**Methods:**

Invasion assays in Matrigel, MMP-2 zymogram analysis, gene microarray analysis and mammary isografts were used to characterize the invasive and proliferative function of cells expressing PDK1. Tissue microarray analysis of human breast cancers was used to measure PDK1 expression in invasive tumors by IHC.

**Results:**

Enhanced invasion on Matrigel in PDK1-expressing cells was accompanied by increased MMP-2 activity resulting from stabilization against proteasomal degradation. Increased MMP-2 activity was accompanied by elevated levels of MT1-MMP, which is involved in generating active MMP-2. Gene microarray analysis identified increased expression of the ECM-associated genes decorin and type I procollagen, whose gene products are substrates of MT1-MMP. Mammary fat pad isografts of PDK1-expressing cells produced invasive adenocarcinomas. Tissue microarray analysis of human invasive breast cancer indicated that PDK1pSer241 was strongly expressed in 90% of samples.

**Conclusion:**

These results indicate that PDK1 serves as an important effector of mammary epithelial cell growth and invasion in the transformed phenotype. PDK1 mediates its effect in part by MT1-MMP induction, which in turn activates MMP-2 and modulates the ECM proteins decorin and collagen. The presence of increased PDK1 expression in the majority of invasive breast cancers suggests its importance in the metastatic process.

## Background

PDK1 was first identified as a protein-Ser/Thr kinase that linked PI3K to Akt activation in response to growth factor receptor stimulation [1,2]. PDK1 phosphorylates AGC kinases such as Akt [3,4], PKC [5,6] and SGK [7,8] in the activation domain, which is a prerequisite for catalytic activity. PDK1 has been studied extensively with respect to its structure, activity, substrate specificity and cellular localization as a signaling molecule critical in the PI3K pathway [9-12]. Tumorigenesis studies have demonstrated that PDK1-expressing mouse mammary epithelial cells (Comma/PDK1) form adenocarcinomas in syngeneic mice [13], and that transformation was related to increased expression of PKCα and β-catenin activation, and to downregulation of the breast tumor suppressor caveolin-1 [13,14]. PDK1 has been found to serve as an effective therapeutic target for inhibition of glioblastoma growth [15].

Cancer mortality is due largely to distant metastases and subsequent organ failure. Metastasis involves the degradation of the basement membrane and stromal ECM and migration into adjoining blood vessels that results in tumor growth at distant organ sites [16,17]. Degradation of the basement membrane and ECM involve the secretion of several proteases, such as one or more members of the MMP family [18,19]. Among the more than 20 MMPs that have been identified [20], MMP-2 has been described as a negative prognostic marker of metastasis and disease-free interval [21,22]. MMP-2 activation and ECM invasion is regulated in Akt1-expressing cells in part by stabilization against proteasomal degradation independently of transformation [23]. Although PDK1 was shown previously to exhibit tumorigenic activity, direct evidence for its involvement in invasion has not been reported. In the present investigation, we show that expression of PDK1 strongly induced ECM invasion, MT1-MMP levels and MMP-2 activity in mammary epithelial cells that was dependent on PI3K activation. In addition, Comma/PDK1 cells formed invasive adenocarcinomas in syngeneic mice, and was highly expressed in 90% of invasive human breast cancers, suggesting that PDK1 may serve as a prognostic indicator of metastasis, as well as a potential therapeutic target.

## Methods

### Cells, antibodies and plasmids

Comma-1D mouse mammary epithelial cells were obtained from Dr. Robert B. Dickson, Georgetown University. Comma-1D cells were retrovirally transduced with either PDK1 (Comma/PDK1) or empty virus (Comma/vector) [13] and maintained at 37°C under 5% CO_2 _in DMEM/F12 medium supplemented with 5% FBS, 10 ng/ml EGF and 5 μg/ml insulin. Human breast cancer cell lines MDA-MB-231 and MCF-7 were obtained from the Tissue Culture Shared Resource, LCCC. Rabbit polyclonal anti-MMP-2 antibody and LY294002 were purchased from EMD Biosciences (La Jolla, CA), rabbit polyclonal antibodies to PDK1pSer241 and Akt1pSer473 were obtained from Cell Signaling Technology (Beverly, MA), and a rabbit polyclonal antibody against MT1-MMP was purchased from Chemicon International (Temecula, CA). The MMP-2 promoter reporter constructs 'WT' and 'D9' [24] were generously provided by Dr. Etty Benveniste, University of Alabama.

### Boyden chamber assay

Invasion assays were carried out in a 48-well Boyden Chamber (NeuroProbe Inc, Rockville, MD) equipped with an 8 μm polycarbonate membrane coated with 20 μg/ml Matrigel (BD Biosciences, San Diego, CA). Cells were serum-starved for 6 hr, and 50 μl containing 10,000 cells in serum-free medium supplemented with 0.1% BSA were loaded into the upper chamber. The lower chamber contained either medium supplemented with 15% FBS as the chemoattractant or serum-free medium containing 0.1% BSA as a negative control. Cells were incubated at 37°C overnight, fixed in 4% formaldehyde for 15 min and stained with Harris-modified hematoxylin (Fisher Scientific, Pittsburgh, PA). Noninvading cells on the top of the membrane were wiped off using a cotton swab, and invading cells affixed to the underside of the membrane were counted in 5 random areas. An equal number of MDA-MB-231 cells were used as a positive control, and invasion of Comma/PDK1 and Comma/vector cells were expressed as a percentage of the number of MDA-MB-231 cells invading Matrigel.

### Zymography

Cells were grown in 75-cm^2 ^cell culture flasks for 48 hr, washed twice with PBS, and incubated in serum-free DMEM/F12 medium for 24 hr. In some instances, cells were treated with 1 μM lactacystin for 24 hr prior to incubation in serum-free medium [23]. Conditioned medium was collected and concentrated using a YM-30 Centriplus centrifugal filter (*M*_*r *_30,000 cutoff, Amicon, Bedford, MA) as described [23]. Concentrates containing 2 μg protein were loaded onto a 10% polyacrylamide gel containing 0.1% gelatin (Invitrogen Corp., Carlsbad, CA) and separated by SDS-PAGE. After electrophoresis, the gel was renatured in 2.5% Triton X-100 solution at room temperature for 30 min with gentle agitation, equilibrated in developing buffer (50 mM Tris-HCl, pH 7.4; 200 mM NaCl, 5 mM CaCl2, 0.02% Brij35) at room temperature for 30 min with gentle agitation, and incubated overnight at 37°C in fresh developing buffer. Transparent bands of gelatinolytic activity were visualized by staining with 0.5% Coomassie Blue R250. In some instances, samples were treated with 1 mM APMA for 1 hr at 37°C prior to zymogram analysis.

### Luciferase assay

Cells were grown in a 24-well plate (40,000 cells/well) and transfected with 100 ng 'WT' or 'D9' MMP-2 promoter plasmid [24] using Lipofectamine Plus reagent (Invitrogen Corp., Carlsbad, CA). Transfection efficiency was monitored by cotransfection with 100 ng pEGFP-C1 (BD Bioscience, Palo Alto, CA) and ≥ 50% efficiency was observed in both cell lines. Luciferase activity was measured after 24 hr using the Luciferase Assay System (Promega, Madison, WI).

### Cell growth

Cells were seeded into a 96-well plate at 3,000 cells/well in 200 μl growth medium, and cell growth was measured 24–72 hr later by sulforhodamine B (SRB) staining [25]. Briefly, at the time of harvest, cells were fixed by addition of 50 μl cold 50% TCA for 1 hr at 4°C and washed five times with tap water. Cells were stained by addition of 50 μl/well of 0.4% SRB in 1% acetic acid at room temperature for 30 min, and rinsed four times with 1% acetic acid to remove unbound dye. SRB was solubilized in 50 μl of 10 mM Tris base (pH 10.5) for 5 min with agitation, and absorbance at 560 nm read in a Cambridge 750 microplate reader (Cambridge Technology, Inc, Cambridge, MA).

### Anchorage-independent cell growth

Cells were seeded in 200 μl growth medium into a Sigmacote-coated Ultra Low Cluster 96-well plate (Corning Life Sciences, Acton, MA) at 16,000 cells/well. Viable cells were measured 72 hr later using the CellTiter-Glo (Promega) luciferase assay according to the manufacturer's protocol. Briefly, at the time of harvest, an equal volume of CellTiter-Glo reagent was added to each well and the plate incubated at room temperature for 15 min with gentle agitation. An aliquot of 100 μl was taken from each well and chemiluminescence determined in a Berthold MicroLumat Plus luminometer.

### Growth on collagen

A collagen gel solution (0.8 mg/ml) was prepared on ice by addition of 1.8 ml rat tail type I collagen (4.3 mg/ml) (BD Biosciences), 1.8 ml 1.8% NaHCO_3 _and 0.18 ml 10× PBS to 6.3 ml serum-free DMEM/F12 medium. Collagen solution (0.5 ml/well) was added to a 24-well plate and allowed to solidify at room temperature for at least 2 hr. Cells (50,000 cells in 1 ml growth medium) were added to the surface of the collagen and incubated at 37°C in a CO_2 _incubator for 7 days. Cells were photographed using a Nikon SMZ-1500 EPI-Fluorescence Stereoscope, Microscopy and Imaging Shared Resource, LCCC.

### Isograft transplantation

Comma/Vector and Comma/PDK1 cells were transplanted into the cleared mammary fat pad of 3 week old BALB/c mice as described [13]. Eight weeks after transplantation, mice were sacrificed, and isografts were fixed in 10% formalin in PBS, embedded in paraffin, and stained with H&E by the Histopathology and Tissue Shared Resource, LCCC. Tumor lysates were analyzed by western blot for the expression of PDK1 as described [13].

### Western blot analysis

Cell lysates containing 100 μg protein were separated in 10% polyacrylamide gels by SDS-PAGE, blotted onto nitrocellulose (Optitran, Schleicher and Schuell, Keene, NH) and analyzed with Akt1, Akt1pSer473, MT1-MMP and β-actin antibodies. For western analysis of MMP-2, concentrates of conditioned medium containing 2 μg protein were mixed with 5X Laemmli sample buffer under nonreducing conditions at room temperature for 10 min, and separated in 10% polyacrylamide gels by SDS-PAGE. Samples were blotted onto nitrocellulose (Optitran, Schleicher and Schuell) and analyzed with an anti-MMP-2 antibody.

### Immunohistochemistry

Paraffin sections of human malignant and benign breast tumors were obtained from the Histopathology and Tissue Shared Resource, LCCC. Tissue microarrays of invasive human breast cancers were obtained from the Cooperative Breast Cancer Tissue Resource (CBCTR), NIH. Slides consisted of 252 normal and breast cancer samples consisting of 64 cores each of node-negative, node-positive and metastatic cancers, 20 cores of DCIS and 40 cores of normal breast tissue. Slides were baked at 56°C over night, deparaffinized in xylene for 10 min, and rehydrated in 100%, 95% and 70% ethanol for 5 min each. Antigen retrieval was achieved by steaming the slides for 30 min in 1 mM EDTA, pH 8.0. Slides were washed three times in PBS and blocked for 30 min in a buffer containing 1% bovine serum albumin and 5% goat serum in PBS. Slides were incubated at 4°C overnight with rabbit anti-PDK1pSer241 diluted 1:50, washed five times in PBS and incubated with biotinylated secondary antibody for 1 hr. Slides were washed five times in PBS and antigen was visualized using ABC Vectastain and DAB as substrate (Vector Labs, Burlingame, CA). Slides were counterstained with Harris-modified hematoxylin (Fisher Scientific, Pittsburgh, PA) and mounted in Permount. Staining intensity was scored 0, +, ++ or +++ for absent, low, medium or high, respectively.

### Gene microarray

Total RNA was prepared from Comma/PDK1 or Comma/vector cells using Trizol according to the manufacturers' instructions (Invitrogen Corp., Carlsbad, CA). cRNA synthesis was carried out using the Affymetrix protocol with minor modifications as described [26]. Biotin-labeled cRNA was fragmented at 94°C for 35 min and used for hybridization overnight to an Affymetrix MurineGenome U74Av2 GeneChip^® ^representing more than 36,000 mouse genes and EST's by the Macromolecular Analysis Shared Resource. The processed chips were scanned using an Agilent Gene Array scanner, and grid alignment and raw data generation was carried out using Affymetrix GeneChip^® ^5.0 software. Each analysis was repeated three times. The expression of genes that were either increased or decreased at least 2-fold in both experiments were clustered hierarchically.

### qrt-PCR

Total RNA (2 μg) was pre-digested with DNase I (Invitrogen) for 15 min and initiated for cDNA synthesis with superscript II RNaseH reverse transcriptase (Invitrogen) and random primers following the manufacturer's protocol. qrt-PCR was performed in triplicate in an ABI-Prism 7700 sequencing instrument (Applied Biosystems, Foster City, CA) using SYBR green I detection as described [26,27]. The increase in fluorescent signal was associated with exponential formation of PCR product during the linear log phase. The threshold cycle (C_T_) value is the cycle at which a significant increase in the reaction product is first detected. The higher the initial amount of cDNA, the sooner accumulated product is detected in the PCR process, and the lower the C_T _value. The expression of each target gene was normalized to the expression of β-actin and is presented as the ratio of the target gene to β-actin gene calculated by 2^-ΔCt^, where ΔCt = Ct^Target^-Ct^β-actin^. Primers used for qrt-PCR are listed in table 2.

## Results

**Figure 1:**
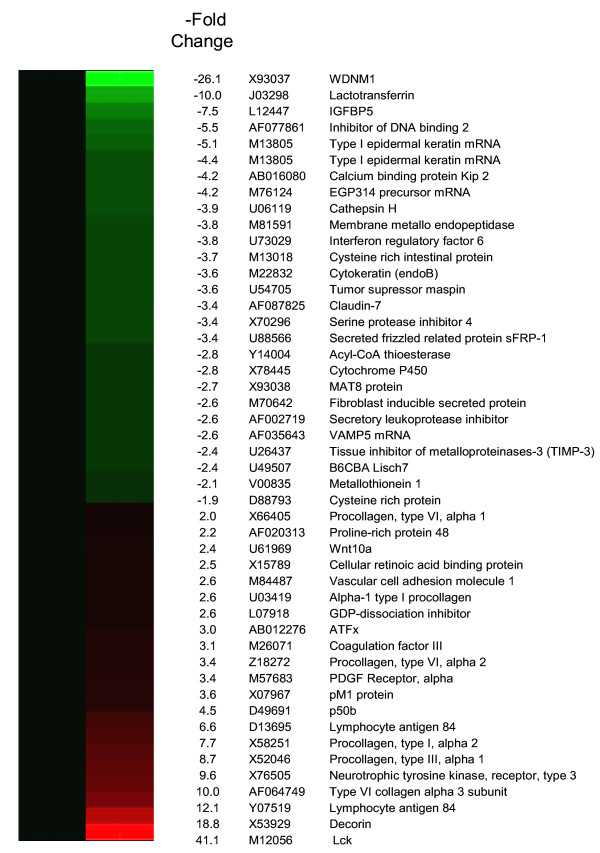
Gene microarray analysis of Comma/PDK1 cells. Total RNA from Comma/vector and Comma/PDK1 cells were used to prepare biotin-labeled cRNA for hybridization as described in Methods. Results are expressed as the -fold change in expression normalized to β-actin expression.

Comma/PDK1 cells were found previously to be tumorigenic in syngeneic mice [13]. To further assess their phenotype, gene expression profiling was used to compare control and PDK1-expressing cells (Fig 1). The expression of 27 genes decreased and 21 genes increased in Comma/PDK1 cells compared to control cells. Among the changes in ECM-related gene expression associated with invasion [28] were an 18-fold increase in decorin, an 11-fold increase in type I procollagen and a 10-fold increase in collagen VI, whose expression has been linked to mammary tumorigenesis [29]. WDNM1, a putative breast cancer metastasis suppressor [30,31], was reduced 26-fold, and the MMP-2 inhibitor, TIMP-3, was also decreased in Comma/PDK1 cells. Several changes in gene expression detected by microarray analysis were confirmed by qrt-PCR (Table 1). There was close agreement between the two methodologies with the exception of the results for Lck. In the latter case, Lck expression was greater in Comma/PDK1 cells as determined by qrt-PCR vs. microarray analysis, which likely reflected the extremely low background and basal expression in control cells as detected by qrt-PCR.

**Figure 2:**
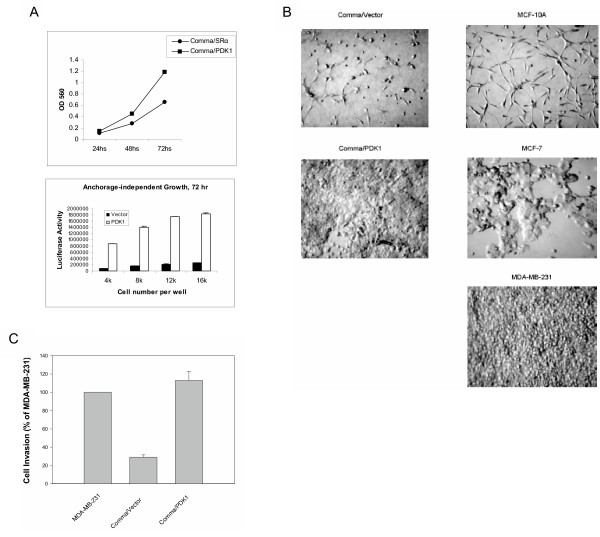
PDK1 increases anchorage-dependent and -independent cell growth and ECM invasion. *A, Top panel*, Cell growth on a plastic substrate was determined after 24–72 hr by SRB staining as described in Methods. Results are expressed as the OD_560 _at each time point. *Lower panel*, Anchorage-independent growth was determined by seeding cells in siliconized plates and determining growth after 72 hr by assay for ATP using luciferase as described in Methods. Results are expressed in units of luciferase activity. *B*, Cells were grown for 7 days on collagen-coated plates. Magnification 50×. *C*, Cells were grown on Matrigel in a Boyden chamber and cells invading through Matrigel were determined. Results are expressed as a percentage of MDA-MB-231 cells invading Matrigel run in parallel as a positive control, and represent the mean ± S.E. of three independent experiments.

Ectopic expression of PDK1 in mammary epithelial cells resulted in increased anchorage-dependent and -independent growth (Fig. 2). Comma/PDK1 cells grew at twice the rate of control cells on a plastic substrate, and even greater differences were noted under conditions of anchorage-independent growth in low adherence silconized plates (Fig 2A). Comma/PDK1 cells also grew avidly on collagen and formed an uneven monolayer with areas of piled up cells, in contrast to control cells which showed little growth under these conditions (Fig. 2B). The growth characteristics of Comma/vector cells mirrored those of human breast epithelial cell line MCF-10A, whereas those of Comma/PDK1 were similar to MCF-7 and MDA-MB-231 breast cancer cells (Fig. 2B). The ability of Comma/PDK1 cells to invade an ECM was also assessed using the Boyden chamber assay with Matrigel as the ECM (Fig. 2C). Comma/PDK1 cells were found to be highly invasive in comparison to control cells, and were equally as invasive as MDA-MB-231 cells that were used as a positive control.

**Figure 3:**
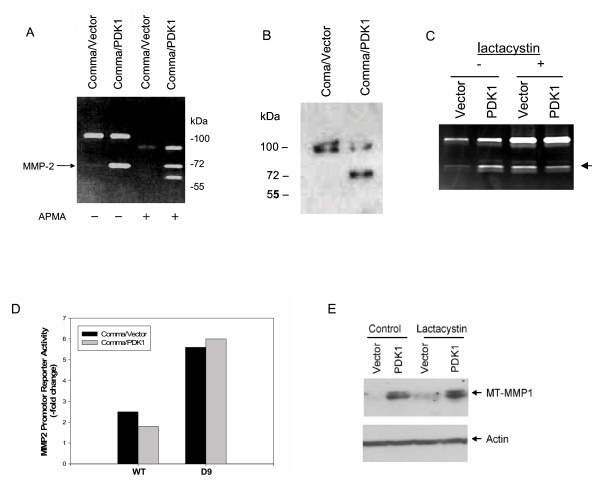
Overexpression of PDK1 increases MMP-2 activity and MT1-MMP. *A*, Comma/Vector and Comma/PDK1 cells were grown and conditioned medium was concentrated and assayed for MMP activity by zymography in the present or absence of 1 mM APMA as described in Methods. Protein markers are indicated by their mass in kDa. *B*, Concentrates of conditioned medium described in *A *containing 2 μg protein were separated under non-reducing conditions by SDS-PAGE in 10% polyacrylamide gels, and blotted onto nitrocellulose and analyzed with an anti-MMP-2 antibody. *C*, Comma/Vector (*Vector*) and Comma/PDK1 (*PDK1*) cells were incubated for 24 hr in the presence and absence of 1 μM lactacystin, followed by incubation in serum-free medium for 24 hr. Samples were assayed by zymography as in *A*. *D*, Cells were transfected with 'WT' or 'D9' MMP-2 promoter plasmids as described in Methods. Luciferase activity was measured after 24 hr and is expressed as the -fold change relative to control cells. Luciferase activity (light units) in control cells with the 'WT' and 'D9' plasmids was 2,970 and 6,520, respectively. *E*, Comma/Vector (*Vector*) and Comma/PDK1 (*PDK1*) cells were treated with or without lactacystin as in *B *and cell lysates analyzed by western blotting for levels of MT1-MMP.

To determine whether invasion was associated with MMP activation, conditioned medium from Comma/PDK1 and Comma/vector cells was concentrated and analyzed by zymography with gelatin as the substrate (Fig. 3). Proteolytic activities of approximately 100 and 72 kDa were present in the conditioned medium from both cell lines, and the 72 kDa species was markedly increased in Comma/PDK1 cells (Fig 3A). Induction of autocatalytic processing with APMA resulted in the disappearance of the 100 kDa activity and appearance of 72 and 66 kDa species (Fig. 3A). Western blotting under non-reducing conditions indicated that the 100 and 72 kDa activities were proMMP-2 and MMP-2, respectively (Fig. 3B). The influence of proteasomal degradation on MMP activity was next tested with the proteasome inhibitor lactacystin (Fig. 3C). Lactacystin markedly increased both proMMP-2 and MMP-2 activities in control cells, but had little or no effect on the already high activity present in the conditioned medium from Comma/PDK1 cells (Fig 3C). These results suggest that PDK1 increased MMP-2 activity in part by attenuating proteasomal degradation. In contrast, PDK1 did not affect reporter gene activity under the control of the MMP-2 promoter [24] as determined with the 139 bp (*D9*) and 1,659 bp (*WT*) MMP-2 promoter regions (Fig. 3D). Cells were also analyzed for MT1-MMP expression since MMP-2 is processed to the catalytically active form by MT1-MMP [32] (Fig. 3E). MT1-MMP levels were markedly increased in PDK1-expressing cells, but were not further increased by lactacystin treatment suggesting that regulation of MT1-MMP expression may be the primary mechanism by which PDK1 regulates MMP-2 activity.

**Figure 4:**
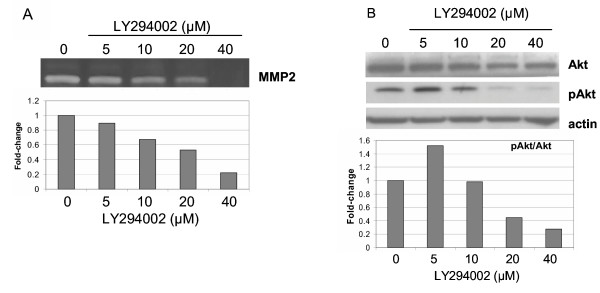
Inhibition of PI3K reduces MMP expression in Comma/PDK1 cells. Cells were treated for 24 hr with 5–40 μM LY294002. *A*, Media was concentrated and assayed for MMP activity by zymography as described in Fig. 3. Bar graph represents a densitometric scan of the zymogram. *B*, Cells were lysed and analyzed by western blotting for levels of Akt, Akt1pS473 and β-actin. Bar graph represents a densitometric scan of the western blots normalized for β-actin levels.

To determine the influence of the PI3K/PDK1 signaling pathway on MMP-2 activation, Comma/PDK1 cells were treated with the PI3K inhibitor LY294002 (Fig. 4). LY294002 reduced 72 kDa MMP-2 activity (Fig. 4A), whereas treatment with either the MEK inhibitor U0126 or the p38 inhibitor SB203580 had no effect (results not shown). In addition, the activity of the downstream PDK1 target, Akt, as determined by Akt1pSer473 expression was inhibited to a similar extent as MMP-2 activity (Fig. 4B).

**Figure 5:**
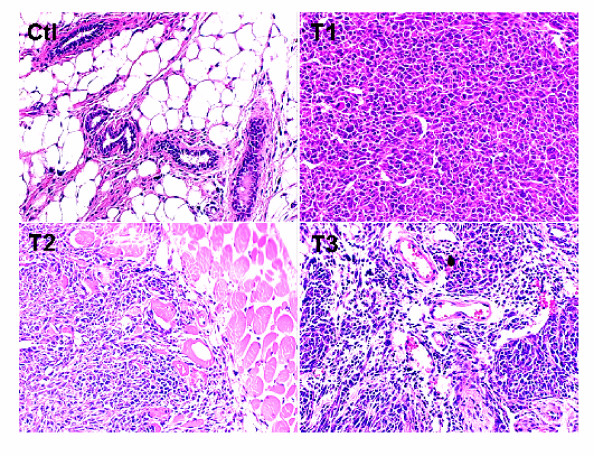
Neoplastic transformation and invasion of isografts of Comma/PDK1 cells. H&E stained paraffin-embedded tissue obtained from isografts of cells transduced with either empty virus (*Ctl*) or PDK1 (*T1, T2, T3*) 8 weeks after transplantation. Mammary isografts of control cells have normal lobuloalveolar ductal morphology with surrounding adipose tissue. Comma/PDK1 isografts produced poorly differentiated adenocarcinomas with solid cords of cells with little gland formation (*T1*), invasion of underlying skeletal muscle (*T2*) and vascularity (*T3*). Magnification: 100×.

The invasive potential of Comma/PDK1 cells *in vivo *was next determined by grafting cells into the cleared mammary fat pad of syngeneic mice (Fig. 5). Comma/PDK1 cells grew into invasive and vascular poorly differentiated adenocarcinomas within 8 weeks after transplantation, in contrast to the normal mammary gland morphology produced by control cells.

**Figure 6:**
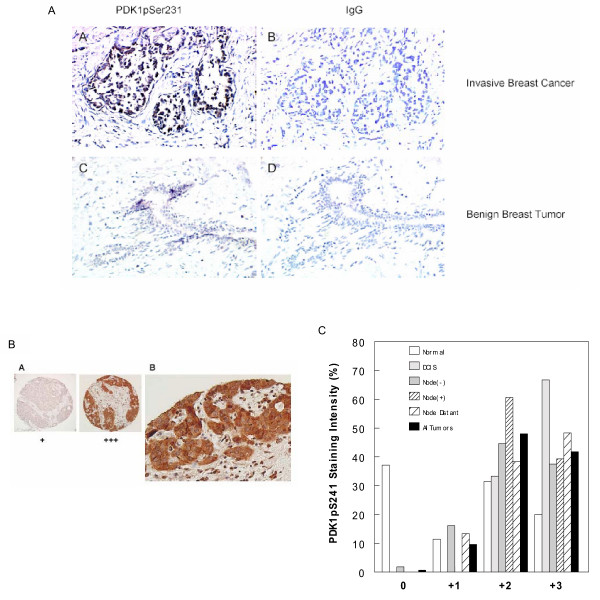
Immunohistochemical detection of PDK1pSer241 in breast cancer specimens. *A*, IHC for PDK1pSer241 in paraffin blocks of benign (*C,D*) and malignant (*A,B*) breast tissue. Staining was conducted with either nonimmune IgG (*B,D*) or a PDK1pSer241 antibody (*A,C*). *B*, Tissue microarray cores of low (+) expressing and high (+++) expressing breast cancers stained for PDK1pSer241. PDK1pSer241 was abundant in both the cytosol and nucleus of the carcinoma. *C*, A bar graph depicting the results in the tissue microarray analysis of 177 evaluable cores expressing either no (0), low (+1), medium (+2) or strong (+3) PDK1pSer241 staining of normal (*normal*), DCIS (*DCIS*), node negative (*node *(-)), node positive (*node *(+)) and metastatic (*node distant*) breast carcinomas.

As a measure of the significance of PDK1 expression in breast cancer invasion, paraffin sections of malignant and benign breast cancers were examined for PDK1pSer241 expression (Fig. 6A). A pilot study determined that paraffin-embedded sections of a ductal breast carcinoma exhibited strong staining for PDK1pSer241, whereas little or no staining occurred in benign breast tumors. To obtain a broader perspective of the significance of PDK1 expression in invasive breast cancer, tissue microarrays of node-negative, node-positive and metastatic breast cancer specimens were assessed for expression of PDK1pSer241 by IHC (Fig. 6B,C). Ninety percent of all tumor samples exhibited moderate to strong staining for PDK1pSer241, with 42% of evaluable samples exhibiting strong expression (74/177 cores). These data further indicate that PDK1 is associated with an invasive phenotype in breast cancer.

## Discussion

The present study demonstrates that PDK1 expression confers a marked growth advantage to mammary epithelial cells that was associated with increased ECM invasion, MMP-2 activity and MT1-MMP expression. Gene microarray analysis additionally identified two known MT1-MMP substrates known to be involved in growth and invasion, decorin and type I procollagen [28]. MT1-MMP expression has been linked to invasive breast cancer and lymph node and distant metastases [33,34], which is consistent with the increased gene expression of two MT1-MMP ECM-related substrates, type I collagen [28,35], and decorin [36] in Comma/PDK1 cells. Thus, the invasive phenotype of Comma/PDK1 cells is closely associated with a heretofore unrecognized phenotype associated with increased MT1-MMP expression and MMP-2 activity.

Array analysis also detected increased collagen VI and reduced WDNM1 and TIMP3 gene expression in PDK1-expressing cells. Collagen VI has been previously linked to mammary tumorigenesis [29], and loss of the tumor suppressor WDNM1 is associated with rat mammary adenocarcinoma [31] and human breast cancer metastasis [30,37]. We have previously linked the tumorigenic phenotype of Comma/PDK1 cells to the disappearance of another breast cancer tumor suppressor, caveolin-1 [14,38]. Thus, reduced expression of two tumor suppressor genes are associated with the PDK1 signaling pathway that may contribute to the invasive and tumorigenic phenotype resulting from unregulated PDK1 expression.

The present study demonstrates that PDK1 markedly increases proliferation, particularly with collagen as a substrate, which is known to induce growth arrest by inhibiting the Ras/Erk pathway and cyclin D1 expression [39]. These results are consistent with activation of cyclin D1 downstream to β-catenin/TCF activation in Comma/PDK1 cells [14]. Increased proliferation may also have occurred through activation of the PI3K/Akt1 axis by PDK1 [10,40,41], which accounts in part for increased cyclin D1 expression [14], mammary hyperplasia in transgenic animal models [42] and invasion [23], but not transformation *per se *[13]. Importantly, the proliferative and invasive characteristics of PDK1-expressing cells were recapitulated *in vivo *as mammary isografts, which attests to the tumorigenic potential of the PDK1 signaling pathway [13,14]. Of note were the similar invasion activity between Comma/PDK1 cells and MDA-MB-231 breast carcinoma cells, which are known for their metastatic behavior [43-45].

MMP-2 activity is an important factor associated with invasion. Overexpression of MMP-2 activity in MDA-MB-231 cells increased invasion *in vitro*, as well as distant metastases in nude mice [46], and high MMP-2 levels was associated with metastatic breast tumors [21,22,47]. PDK1 modulated MMP-2 activity in part through stabilization against proteasomal degradation, a mechanism similar to that described for Akt1 and v-akt [23]. MMP-2 expression is linked to IGF-I signaling [48] and MT1-MMP, which activates the proenzyme form of MMP-2, and is upregulated via the PI3K/Akt1 pathway [32]. These findings are consistent with the increased expression of MT1-MMP in Comma/PDK1 cells since Akt is a downstream effector of PDK1, as well as the ability of LY294002 to block MMP-2 activation and inhibit Akt activity. Our finding that PDK1 is activated in a large percentage of invasive human breast cancers further suggests the importance of the PDK1 signaling pathway to the metastatic phenotype.

## Conclusion

The present study demonstrates that PDK1 expression in mammary epithelial cells confers not only a growth advantage, but also an invasive phenotype characterized by increased MMP-2 activity and MT1-MMP expression. These results further define the tumorigenic and invasive processes elicited by PDK1, and suggest a fundamental new role for the PDK1 pathway in breast cancer growth and metastasis.

## Abbreviations

APMA, 4-aminophenylmercuric acetate; Comma/PDK1, Comma-1D mouse mammary epithelial cells stably expressing PDK1; DCIS, ductal carcinoma *in situ*; ECM, extracellular matrix; IHC, immunohistochemistry; LCCC, Lombardi Comprehensive Cancer Center; MMP, matrix metalloproteinase; MT1-MMP, membrane-associated matrix metalloproteinase-1; PDK1, 3-phosphoinositide-dependent protein kinase-1; PI3K, phosphatidylinositol 3-kinase; PKC, protein kinase C; qrt-PCR, quantitative real-time PCR; SRB, sulforhodamine B.

## Competing interests

The author(s) declare that they have no competing interests.

## Authors' contributions

RIG was responsible for data analysis, drafting the manuscript and the overall direction of these studies. ZX and YY participated in the study design and gene array analysis. ZX and HY performed cell growth, western blot and MMP analyses. ZX conducted immunohistochemical analysis of breast tumors. RB conducted the qrt-PCR analysis and XZ and RIG performed the isografts. All authors read and approved the manuscript.

## Pre-publication history

The pre-publication history for this paper can be accessed here:



## Figures and Tables

**Table 1:** PDK1-induced gene expression by qrt-PCR

Cell line	WDNM1	TIMP3	Lck	Collagen VI
Comma/vector	144.9 ± 11.0	156.5 ± 6.0	0.005 ± 0.001	0.56 ± 0.02
Comma/PDK1	3.1 ± 0.1	84.6 ± 2.3	43.6 ± 1.3	4.4 ± 0.3
-fold change	-46.7	-1.8	8,720	7.9
-fold change in microarray	-26.1	-2.4	41.1	10.0

Values are expressed as the absolute mRNA levels present in Comma/PDK1 and Comma/vector cells in arbitrary units relative to the expression of β-actin. Each result is the mean ± S.E. of 3 determinations. The relative values from gene microarray analysis are shown for comparison

**Table 2:** 

Genes	Forward primer (5' → 3')	Reverse primer (5' → 3')
WDNM1	AGACAGCCACAGTCTTTGTTCTG	GATCCATCTCCTGTGCATCGTTC
TIMP3	CCC TTT GGC ACT CTG GTC TA	TCC TGT GTA CAT CTT GCC TTC ATA
Lck	CAA GAA TCT GAG CCG TTA GGA	GGA GAT GTA GAA GCC ACC GTT GT
Collagen VI	CCA AAC CCA CGG GAC CTC AAA	CTC ACT GGC AAA GCT GTA GAC C
β-Actin	AGA GGG AAA TCG TGC GTG AC	CAA TAG TGA TGA CCT GGC CGT

## References

[B1] Alessi DR, Deak M, Cassamayor A, Caudwell FB, Morrice N, Norman DG, Gaffney P, Reese CB, MacDougall CN, Harbison D, Ashworth A, Bownes M (1997). 3-Phosphoinositide-dependent protein kinase-1 (PDK1): structural and functional homology with the Drosophila DSTPK61 kinase. Curr Biol.

[B2] Stephens L, Anderson K, Stokoe D, Erdjument-Bromage H, Painter GF, Holmes AB, Gaffney PR, Reese CB, McCormick F, Tempst P, Coadwell J, Hawkins PT (1998). Protein kinase B kinases that mediate phosphatidylinositol 3,4,5- trisphosphate-dependent activation of protein kinase B. Science.

[B3] Filippa N, Sable CL, Hemmings BA, Van Obberghen E (2000). Effect of phosphoinositide-dependent kinase 1 on protein kinase B translocation and its subsequent activation. Mol Cell Biol.

[B4] Williams MR, Arthur JS, Balendran A, van der Kaay J, Poli V, Cohen P, Alessi DR (2000). The role of 3-phosphoinositide-dependent protein kinase 1 in activating AGC kinases defined in embryonic stem cells. Curr Biol.

[B5] Cenni V, Doppler H, Sonnenburg ED, Maraldi N, Newton AC, Toker A (2002). Regulation of novel protein kinase C epsilon by phosphorylation. Biochem J.

[B6] Dutil EM, Toker A, Newton AC (1998). Regulation of conventional protein kinase C isozymes by phosphoinositide-dependent kinase 1 (PDK-1). Curr Biol.

[B7] Kobayashi T, Deak M, Morrice N, Cohen P (1999). Characterization of the structure and regulation of two novel isoforms of serum- and glucocorticoid-induced protein kinase. Biochem J.

[B8] Park J, Leong ML, Buse P, Maiyar AC, Firestone GL, Hemmings BA (1999). Serum and glucocorticoid-inducible kinase (SGK) is a target of the PI 3- kinase-stimulated signaling pathway. Embo J.

[B9] Toker A, Newton AC (2000). Cellular signaling: pivoting around PDK-1. Cell.

[B10] Vanhaesebroeck B, Alessi DR (2000). The PI3K-PDK1 connection: more than just a road to PKB. Biochem J.

[B11] Belham C, Wu S, Avruch J (1999). Intracellular signalling: PDK1--a kinase at the hub of things. Curr Biol.

[B12] King CC, Newton AC (2004). The adaptor protein Grb14 regulates the localization of 3-phosphoinositide dependent kinase-1 (PDK-1). J Biol Chem.

[B13] Zeng X, Xu H, Park BK, Glazer RI (2002). Transformation of  mammary epithelial cells by 3-phosphoinositide-dependent protein kinase-1 (PDK1) is associated with the induction of protein kinase Ca. Cancer Res.

[B14] Xie Z, Zeng X, Waldman T, Glazer RI (2003). Transformation of mammary epithelial cells by 3-phosphoinositide- dependent protein kinase-1 activates beta-catenin and c-Myc, and down-regulates caveolin-1. Cancer Res.

[B15] Flynn P, Wongdagger M, Zavar M, Dean NM, Stokoe D (2000). Inhibition of PDK-1 activity causes a reduction in cell proliferation and survival. Curr Biol.

[B16] Engbring JA, Kleinman HK (2003). The basement membrane matrix in malignancy. J Pathol.

[B17] Itoh Y, Nagase H (2002). Matrix metalloproteinases in cancer. Essays Biochem.

[B18] Hornebeck W, Maquart FX (2003). Proteolyzed matrix as a template for the regulation of tumor progression. Biomed Pharmacother.

[B19] Westermarck J, Kahari VM (1999). Regulation of matrix metalloproteinase expression in tumor invasion. Faseb J.

[B20] Visse R, Nagase H (2003). Matrix metalloproteinases and tissue inhibitors of metalloproteinases: structure, function, and biochemistry. Circ Res.

[B21] Leppa S, Saarto T, Vehmanen L, Blomqvist C, Elomaa I (2004). A high serum matrix metalloproteinase-2 level is associated with an adverse prognosis in node-positive breast carcinoma. Clin Cancer Res.

[B22] Sheen-Chen SM, Chen HS, Eng HL, Sheen CC, Chen WJ (2001). Serum levels of matrix metalloproteinase 2 in patients with breast cancer. Cancer Lett.

[B23] Park BK, Zeng X, Glazer RI (2001). AKT1 induces extracellular matrix invasion and MMP-2 activity in mouse mammary epithelial cells. Cancer Res.

[B24] Qin H, Sun Y, Benveniste EN (1999). The transcription factors Sp1, Sp3, and AP-2 are required for constitutive matrix metalloproteinase-2 gene expression in astroglioma cells. J Biol Chem.

[B25] Skehan P, Storeng R, Scudiero D, Monks A, McMahon J, Vistica D, Warren JT, Bokesch H, Kenney S, Boyd MR (1990). New colorimetric cytotoxicity assay for anticancer-drug screening. J Natl Cancer Inst.

[B26] Yin Y, Bai R, Russell RG, Beildeck ME, Xie Z, Kopelovich L, Glazer RI (2005). Characterization of medroxyprogesterone and DMBA-induced multilineage mammary tumors by gene expression profiling. Mol Carcinog.

[B27] Livak KJ, Schmittgen TD (2001). Analysis of relative gene expression data using real-time quantitative PCR and the 2(-Delta Delta C(T)) Method. Methods.

[B28] Sato H, Takino T, Miyamori H (2005). Roles of membrane-type matrix metalloproteinase-1 in tumor invasion and metastasis. Cancer Sci.

[B29] Iyengar P, Combs TP, Shah SJ, Gouon-Evans V, Pollard JW, Albanese C, Flanagan L, Tenniswood MP, Guha C, Lisanti MP, Pestell RG, Scherer PE (2003). Adipocyte-secreted factors synergistically promote mammary tumorigenesis through induction of anti-apoptotic transcriptional programs and proto-oncogene stabilization. Oncogene.

[B30] Steeg PS (1989). Search for metastasis suppressor genes. Invasion Metastasis.

[B31] Dear TN, Ramshaw IA, Kefford RF (1988). Differential expression of a novel gene, WDNM1, in nonmetastatic rat mammary adenocarcinoma cells. Cancer Res.

[B32] Zhang D, Brodt P (2003). Type 1 insulin-like growth factor regulates MT1-MMP synthesis and tumor invasion via PI 3-kinase/Akt signaling. Oncogene.

[B33] Ishigaki S, Toi M, Ueno T, Matsumoto H, Muta M, Koike M, Seiki M (1999). Significance of membrane type 1 matrix metalloproteinase expression in breast cancer. Jpn J Cancer Res.

[B34] Ueno H, Nakamura H, Inoue M, Imai K, Noguchi M, Sato H, Seiki M, Okada Y (1997). Expression and tissue localization of membrane-types 1, 2, and 3 matrix metalloproteinases in human invasive breast carcinomas. Cancer Res.

[B35] Ohuchi E, Imai K, Fujii Y, Sato H, Seiki M, Okada Y (1997). Membrane type 1 matrix metalloproteinase digests interstitial collagens and other extracellular matrix macromolecules. J Biol Chem.

[B36] Li Y, Aoki T, Mori Y, Ahmad M, Miyamori H, Takino T, Sato H (2004). Cleavage of lumican by membrane-type matrix metalloproteinase-1 abrogates this proteoglycan-mediated suppression of tumor cell colony formation in soft agar. Cancer Res.

[B37] Kluger HM, Kluger Y, Gilmore-Hebert M, DiVito K, Chang JT, Rodov S, Mironenko O, Kacinski BM, Perkins AS, Sapi E (2004). cDNA microarray analysis of invasive and tumorigenic phenotypes in a breast cancer model. Lab Invest.

[B38] Williams TM, Medina F, Badano I, Hazan RB, Hutchinson J, Muller WJ, Chopra NG, Scherer PE, Pestell RG, Lisanti MP (2004). Caveolin-1 gene disruption promotes mammary tumorigenesis and dramatically enhances lung metastasis in vivo. Role of Cav-1 in cell invasiveness and matrix metalloproteinase (MMP-2/9) secretion. J Biol Chem.

[B39] Fassett JT, Tobolt D, Nelsen CJ, Albrecht JH, Hansen LK (2003). The role of collagen structure in mitogen stimulation of ERK, cyclin D1 expression, and G1-S progression in rat hepatocytes. J Biol Chem.

[B40] Aoki M, Batista O, Bellacosa A, Tsichlis P, Vogt PK (1998). The akt kinase: molecular determinants of oncogenicity. Proc Natl Acad Sci U S A.

[B41] Scheid MP, Marignani PA, Woodgett JR (2002). Multiple phosphoinositide 3-kinase-dependent steps in activation of protein kinase B. Mol Cell Biol.

[B42] Ackler S, Ahmad S, Tobias C, Johnson MD, Glazer RI (2002). Delayed mammary gland involution in MMTV-AKT1 transgenic mice. Oncogene.

[B43] Sheng S, Carey J, Seftor EA, Dias L, Hendrix MJ, Sager R (1996). Maspin acts at the cell membrane to inhibit invasion and motility of mammary and prostatic cancer cells. Proc Natl Acad Sci U S A.

[B44] Thompson EW, Reich R, Shima TB, Albini A, Graf J, Martin GR, Dickson RB, Lippman ME (1988). Differential regulation of growth and invasiveness of MCF-7 breast cancer cells by antiestrogens. Cancer Res.

[B45] Brunner N, Thompson EW, Spang-Thomsen M, Rygaard J, Dano K, Zwiebel JA (1992). lacZ transduced human breast cancer xenografts as an in vivo model for the study of invasion and metastasis. Eur J Cancer.

[B46] Tester AM, Waltham M, Oh SJ, Bae SN, Bills MM, Walker EC, Kern FG, Stetler-Stevenson WG, Lippman ME, Thompson EW (2004). Pro-matrix metalloproteinase-2 transfection increases orthotopic primary growth and experimental metastasis of MDA-MB-231 human breast cancer cells in nude mice. Cancer Res.

[B47] Hao X, Sun B, Hu L, Lahdesmaki H, Dunmire V, Feng Y, Zhang SW, Wang H, Wu C, Fuller GN, Symmans WF, Shmulevich I, Zhang W (2004). Differential gene and protein expression in primary breast malignancies and their lymph node metastases as revealed by combined cDNA microarray and tissue microarray analysis. Cancer.

[B48] Long L, Navab R, Brodt P (1998). Regulation of the Mr 72,000 type IV collagenase by the type I insulin-like growth factor receptor. Cancer Res.

